# pSY153-MDR, a p12969-DIM-related mega plasmid carrying *bla*_IMP-45_ and *armA*, from clinical *Pseudomonas putida*

**DOI:** 10.18632/oncotarget.19496

**Published:** 2017-07-22

**Authors:** Min Yuan, Hai Chen, Xiong Zhu, Jiao Feng, Zhe Zhan, Defu Zhang, Xia Chen, Xiaofei Zhao, Jinxing Lu, Jianguo Xu, Dongsheng Zhou, Juan Li

**Affiliations:** ^1^ State Key Laboratory of Infectious Diseases Prevention and Control, Collaborative Innovation Center for Diagnosis and Treatment of Infectious Disease, National Institute for Communicable Disease Control and Prevention, Chinese Center for Disease Control and Prevention, Beijing 102206, China; ^2^ Department of Clinical Laboratory, People’s Hospital of Sanya, Hainan 572000, China; ^3^ State Key Laboratory of Pathogen and Biosecurity, Beijing Institute of Microbiology and Epidemiology, Beijing 100071, China

**Keywords:** *Pseudomonas putida*, multidrug resistant, IMP-45, armA, pSY153-MDR

## Abstract

This work characterized mega plasmid pSY153-MDR, carrying *bla*_IMP-45_ and *armA*, from a multidrug-resistant (MDR) *Pseudomonas putida* isolate from the urine of a cerebral infarction patient in China. The backbone of pSY153-MDR was closely related to *Pseudomonas* plasmids p12969-DIM, pOZ176, pBM413, pTTS12, and pRBL16, and could not be assigned to any of the known incompatibility groups. The accessory modules of pSY153-MDR were composed of 10 individual insertion sequence elements and two different MDR regions, and differed dramatically from the above plasmids. Fifteen non-redundant resistance markers were identified to be involved in resistance to at least eight distinct classes of antibiotics. All of these resistance genes were associated with mobile elements, and were embedded within the two MDR regions. *bla*_IMP-45_ and *armA* coexisted in a Tn*1403*–Tn*1548* region, which was generated from homologous recombination of Tn*1403*- and Tn*1548*-like transposons. The second copy of *armA* was a component of the IS*CR28*–*armA*–∆IS*CR28* structure, representing a novel *armA* vehicle. This vehicle was located within In48, which was related to In363 and In1058. Data presented here provide a deeper insight into the evolutionary history of SY153, especially in regard to how it became extensively drug-resistant.

## INTRODUCTION

*Pseudomonas putida* is a non-fermentative Gram-negative bacillus belonging to the fluorescent group of the genus *Pseudomonas* [1]. It is an opportunistic human pathogen, responsible for nosocomial infections in immunocompromised patients and in those with catheter or indwelling devices. *P. putida* infection usually presents as bacteremia, urinary tract infection, or pneumonia [1, 2].

Worldwide, the increasing emergence of carbapenem-resistant bacteria, including *Pseudomonas* strains, has threatened the efficacy of carbapenems in the treatment of refractory infections [3]. Metallo-β-lactamases (MBLs) are a group of β-lactamases that can hydrolyze all β-lactams, including carbapenems, except aztreonam [3]. Genes encoding these MBLs are generally associated with integrons and transposons, and often coexist with genes conferring resistance to other classes of antibiotics [4]. This association results from co-selection under the pressure of multiple antibiotics, and leads to multidrug resistance (MDR) in *Pseudomonas* strains [4].

Production of MBLs, such as IMPs [5–7], VIMs [5, 8, 9], NDM-1 [10], and DIM-2 [11], has been identified to account for carbapenem resistance in *P. putida*. Previous studies of these MBL-producing *P. putida* strains have been confined to PCR detection and/or sequencing of *bla* genes and their genetic environments, except for that of the *bla*_DIM-2_-carrying plasmid p12969-DIM from a clinical MDR *P. putida* isolate, in which the plasmid was fully sequenced. The backbone of p12969-DIM was genetically closely related to *bla*_IMP-9_-carrying *Pseudomonas aeruginosa* plasmid pOZ176; however, the two plasmids contained dramatically different accessory regions, particularly those containing resistance genes [11, 12]. *bla*_DIM-2_ and *bla*_IMP-9_ in p12969-DIM and pOZ176 were embedded in integrons In1224 and In244, respectively, which were further associated with two different Tn*21* subgroup transposons belonging to the Tn*3* family [11, 12].

The current study presents the second fully-sequenced MBL-encoding plasmid, designated pSY153-MDR, from clinical *P. putida*. pSY153-MDR was a 468.2-kb mega plasmid, and carried *bla*_IMP-45_ (encoding carbapenem resistance) and *armA* (encoding aminoglycoside resistance), as well as additional markers involved in resistance to β-lactams, quinolones, macrolides, tetracyclines, amphenicols, quaternary ammonium compounds, sulphonamides, trimethoprim, and rifampicin. Twelve accessory modules, which especially included two novel MDR regions containing all of the above resistance genes, were inserted at different sites of the pSY153-MDR backbone.

## RESULTS AND DISCUSSION

### General features of *P. putida* SY153

PCR results demonstrated that strain SY153 harbored *bla*_IMP_, *bla*_OXA_, and *armA*, but none of the rest *bla* and 16S rRNA methylase genes screened for in this analysis, with the first two genes being confirmed as *bla*_IMP-45_ and *bla*_OXA-1_ by genomic sequencing. IMP-45 was closely related to IMP-9, with a single Ser214Gly variation, while the Carba NP test showed that SY153 had class B carbapenemase activity (data not shown). Strain SY153 was highly resistant to penicillins, cephalosporins, carbapenems, aminoglycosides, fluoroquinolones, tetracycline, trimethoprim/sulfamethoxazole, and chloramphenicol, but remained intermediately resistant to aztreonam. In addition, greatly elevated MIC values were observed for macrolides, rifampin, and nitrofurantoin, for which resistance breakpoints have not been established for *P. putida* (Supplementary Table 1). Repeated attempts at conjugation failed to transfer the *bla*_IMP-45_ marker from SY153 to *E. coli* J53 and *P. aeruginosa* PAO1.

### Overview of pSY153-MDR

Plasmid pSY153-MDR had a closed circular DNA sequence, 468,170 kb in length, with a mean G+C content of 56.6%. There were 558 predicted open reading frames (ORFs), 68.6% of which encoded hypothetical proteins (Supplementary Figure 1). The molecular structure of pSY153-MDR could be divided into separate accessory modules that were defined as the acquired DNA regions associated with mobile elements, and the remaining backbone regions.

The pSY153-MDR backbone, 385 kb in length, was closely related (>98% nucleotide identity over >86% of the backbone regions) to five plasmids deposited in GenBank, namely pBM413 (a 423-kb *bla*_IMP-45_-harboring plasmid from *P. aeruginosa*; accession number CP016215), pOZ176 [12], pRBL16 (a 370-kb plasmid without antibiotic resistance genes from organic pollutant degradant *Pseudomonas citronelloli*; accession number CP015879), pTTS12 (a 584-kb solvent-resistance plasmid from *P. putida*; accession number CP015879) [13], and p12969-DIM [11] (last accessed December 1, 2016). *repA* (replication initiation protein of unknown incompatibility group) and *parB2*-*parAB* (partition) constituted the sole replication/stability system in pSY153-MDR. Together with *pil* (pilus assemble) and *che* (chemotaxis), this replication/stability system was found in all five plasmids. Two resistance loci, namely *nfxB*–*mexCD*–*oprJ* (resistance-nodulation-division (RND)-type multidrug efflux pump) and *ter* (tellurium resistance), were identified in the pSY153-MDR backbone. *nfxB*–*mexCD*–*oprJ* was also found in pBM413 and pDIM-12969, while the *ter* locus was also identified in plasmids pBM413, pRBL16, and pTTS12.

The accessory regions of pSY153-MDR were composed of four separate copies of each of the insertion sequence (IS) elements IS*1491* and IS*Ppu29*, single copies of IS*1411* and IS*Ppu30*, and two novel MDR regions, designated MDR-1 and MDR-2 (55.7 kb and 9.2 kb in length, respectively). These 12 accessory modules were inserted at different sites of the pSY153-MDR backbone, and all 10 individual IS elements were flanked by direct repeats (DRs; target site duplication signals of transposition). In contrast, only two accessory modules (an IS*Ppu23* element and a MDR region) were identified in p12969-DIM. Compared with p12969-DIM, pSY153-MDR had undergone much more massive insertions of foreign genetic contents, and showed a higher degree of genomic plasticity.

**Figure 1:**
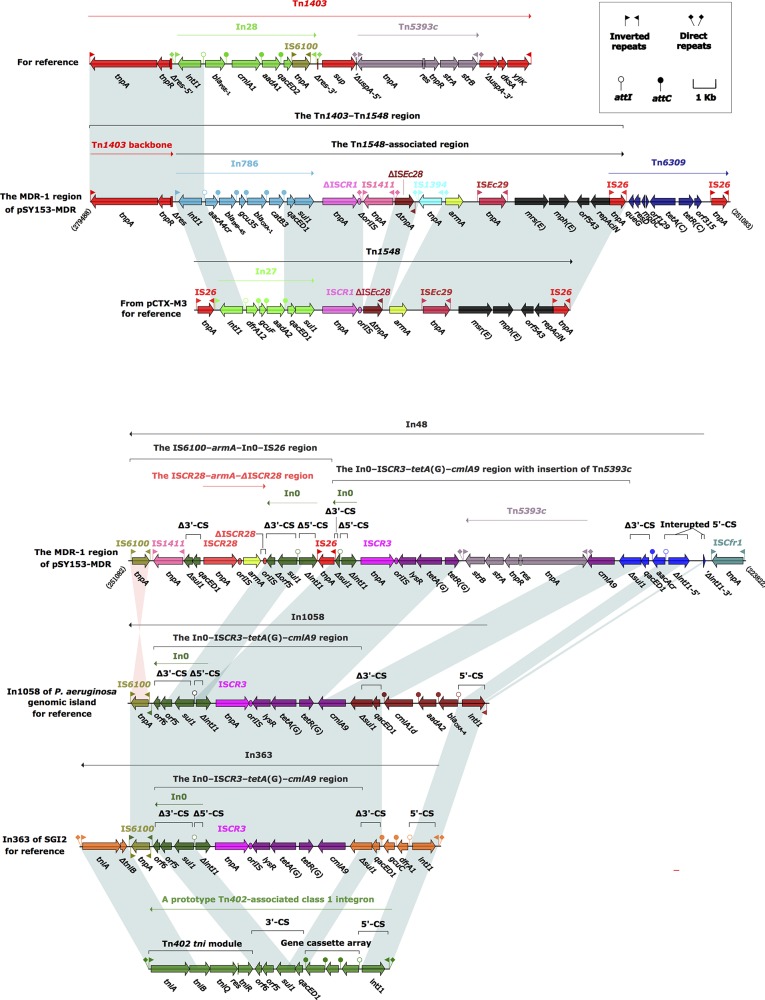
The MDR-1 region from pSY153-MDR, and comparison with related regions Genes are denoted by arrows. Genes, mobile elements and other features are colored based on function classification. Shading denotes regions of homology (>95% nucleotide identity). Numbers in brackets indicate the nucleotide positions within plasmid pSY153-MDR. The accession numbers of Tn*1403*, Tn*1548*, In1058, and In363 for references are AF313472, AF550415, KJ463833, and AY963803, respectively.

The MDR-1 region of pSY153-MDR was organized sequentially, as follows: the Tn*1403*–Tn*1548* region, Tn*6309*, In48, and IS*Cfr1* (Figure 1). Either the MDR-1 region of pSY153-MDR or the MDR region of p12969-DIM was inserted immediately downstream of *nfxB*–*mexCD*–*oprJ,* indicating a “hotspot” for insertion of external genetic material in these two closely related plasmids.

### The Tn*1403*–Tn*1548* region and Tn*6309* from the MDR-1 region

Tn*1403* is a Tn*3*-family transposon initially identified in a clinical *P. aeruginosa* isolate in the United States in the 1970s [14]. It has a core backbone consisting of IRL (inverted repeat left), *tnpA* (transposase), *tnpR* (resolvase), *res* (resolution site), *sup* (sulfate permease), *uspA* (universal stress protein), *dksA* (RNA polymerase-binding transcription factor), *yjiK* (hypothetical protein), and IRR (inverted repeat right), with insertions of In28 and Tn*5393c* into *res* and *dksA*, respectively. Tn*1403* and its close derivatives Tn*6060*, Tn*6061*, Tn*6217*, Tn*6249*, and Tn*6286* in *Pseudomonas* often contain different insertions of foreign material (such as integrons and transposons), serving as important vehicles for transmission of resistance genes [11, 12, 15–17].

Tn*154*8, originally characterized in pCTX-M3 from *Citrobacter freundii*, is an IS*26*-flanked composite transposon without flanking DRs, and displays a modular structure consisting of IS*26*–In27–IS*CR1*–∆IS*Ec28*–*armA*–IS*Ec29*–*msr*(E)–*mph*(E) –*orf543*–*repAciN*–IS*26* [18]. Tn*1548* and its variants mainly differ from each other by insertion of distinct integrons or integron-like elements between IS*26* and IS*CR1*, and are responsible for dissemination of the aminoglycoside resistance gene *armA*, the macrolide resistance operon *msr*(E)–*mph*(E), and other integron-borne resistance genes commonly found among *Enterobacteriaceae* and *Acinetobacter* species [19, 20].

The Tn*1403*–Tn*1548* region of pSY153-MDR was composed of Tn*1403* backbone remnant IRL–*tnpAR*–∆*res*, and a 19.0-kb Tn*1548*-associated region. Compared with Tn*1548*, the Tn*1548*-associated region of pSY153-MDR had undergone several evolutionary events: i) deletion of the IS*26* at the 5’-flank; ii) replacement of In27 with In786; and iii) insertion of IS*1411* and IS*1394* upstream of ΔIS*Ec28* and *armA*, respectively. In786 consisted of an inverted repeat at the integrase end (IRi), a 5’-conserved segment (5’-CS:*intl1* (integrase)–*attI1* (IntI1-recognizing recombination site)), a gene cassette array (GCA; organized as *aacA4cr* (aminoglycoside and quinolone resistance), *bla*_IMP-45_, *gcu35* (unknown function), *bla*_OXA-1_ (β-lactam resistance), *catB3* (chloramphenicol resistance), and a 3’-CS (*qacED1* (quaternary ammonium compound resistance)–*sul1* (sulfonamide resistance)), but lacked an inverted repeat at the *tni* end (IRt). The Tn*1403*–Tn*1548* region was likely generated from homologous recombination between the Tn*1403*-like and Tn*1548*-like transposons, with In786 as the common component.

Tn*6309* is an IS*26*-flanked composite transposon carrying class C tetracycline resistance module *tetA*(C) (class C tetracycline efflux protein)-*tetR*(C) (transcriptional repressor of *tetA*), and has been identified in genomic island Sm1-MDRGI from *Stenotrophomonas maltophilia* [21], and in sequenced plasmids pP10164-3 from *Leclercia adecarboxylata* P10164 [22], pB3 plasmids from *Pseudomonas* sp. GFP1 [23], pNDM-116-14 (accession number LN831184) from *Vibrio cholerae* 116-14, and pKZ3 from an uncultured bacterium [24]. Tn*6309* from pP10164-3 and pB3 is bordered by 9-bp and 5-bp DRs, respectively, indicating that its mobilization into these two plasmids occurred via IS*26*-mediated replicative transposition. No DRs were associated with Tn*6309* in pSY153-MDR and, moreover, Tn*6309* and the Tn*1548-*associated region overlapped by one of their terminal IS*26* elements, suggesting that the connection of Tn*6309* and the Tn*1548-*associated region was promoted by IS*26*-mediated homologous recombination, rather than Tn*6309* transposition.

### In48 from the MDR-1 region

Class 1 integrons are frequently associated with the core transposition module *tniABQ*–*res*–*tniR* (designated *tni*) of Tn*402*, and display a prototype hybrid structure consisting of IRi, 5’-CS, GCA, 3’-CS, *tni*, and IRt [25].

In363 was initially characterized in the SGI2 resistance island from *Salmonella enteric* serovar Emerk [26], and is organized as follows: IRi, 5’-CS, GCA (*dfrA1* (dihydrofolate resistance)–*gcuC* (unknown function)), Δ3’-CS (*qacED1*–Δ*sul1*), the In0 (an empty class 1 integron)–IS*CR3*–*tetA*(G) (class G tetracycline resistance)–*cmlA9* (chloramphenicol resistance) region, and IRi–IS*6100*–IRt–Δ*tni* (Δ*tniB*–*tniA*)–IRt. In363 is bracketed by 5-bp DRs, indicating that its mobilization into SGI2 was a transposition event. In363 differed from the prototype class 1 integron by: i) insertion of the In0–IS*CR3*–*tetA*(G)–*cmlA9* region within the 3’-CS of In363, likely resulting from homologous recombination based on the common 3’-CS region shared by In0 and ancestral In363; and ii) truncation of *tni* by the insertion of IS*6100*, generating the IRi–IS*6100*–IRt–Δ*tni*–IRt structure.

In1058, a close variant of In363, was present in the *bla*_VIM-2_-carrying genomic island from a MDR *P. aeruginosa* isolate [27]. It carried a GCA consisting of *bla*_OXA-4_ (β-lactam resistance)–*aadA2* (aminoglycoside resistance)–*cmlA1d* (chloramphenicol resistance). Other than their differing GCA contents, In1058 had lost the 3’-terminal IRt–Δ*tni*–IRt region relative to In363.

Compared with In1058, In48 had at least five major modular differences: i) In48 carried a single-gene (*aacA4cr*) cassette; ii) IRi was deleted as a result of the connection of IS*Ppu31* to *intI1*; iii) *intI1* was interrupted by a cryptic 617-bp sequence; iv) Tn*5393c* was inserted between *tetA*(G) and *cmlA9* of the In0–IS*CR3*–*tetA*(G)–*cmlA9* region; and v) the IS*6100*–*armA*–In0–IS*26* region was inserted within *sul1* of the In0–IS*CR3*–*tetA*(G)–*cmlA9* region.

The IS*6100*–*armA*–In*0*–IS*26* region was bordered by IS*26* and IS*6100*, both of which belonged to the IS*6* family, and possess almost identical 14-bp terminal inverted repeats. As such, this region might utilize a mechanism of replicative transposition for mobility similar to that used by the IS*26*-flanked composite transposons [28]. In addition, homologous recombination based on the common In0 sequence shared by the IS*6100*–*armA*–In0–IS*26* region and the In0–IS*CR3*–*tetA*(G)–*cmlA9* region might also promote the mobilization of the former region into In48.

The four copies of In0 found in In363, In1058, and In48 had an identical 37-bp *attI1* site, which was the 5’-terminal segment of the intact *attI1*, and lacked the symmetrical structure. This would render these In0s incapable of capturing gene cassette(s) via site-specific recombination.

There were also two copies of *armA* in pSY153-MDR. One copy was located in the Tn*1403*–Tn*1548* region that was a Tn*154*8 variant. The *armA* genes from Tn*1548* and its variants are generally bordered by ∆IS*Ec28* and IS*Ec29* [19, 20]. The second copy of *armA* was flanked by two copies of IS*CR28*, displaying an IS*CR28*–*armA*–∆IS*CR28* structure that represented a novel *armA* vehicle.

### The MDR-2 region of pSY153-MDR

**Figure 2:**
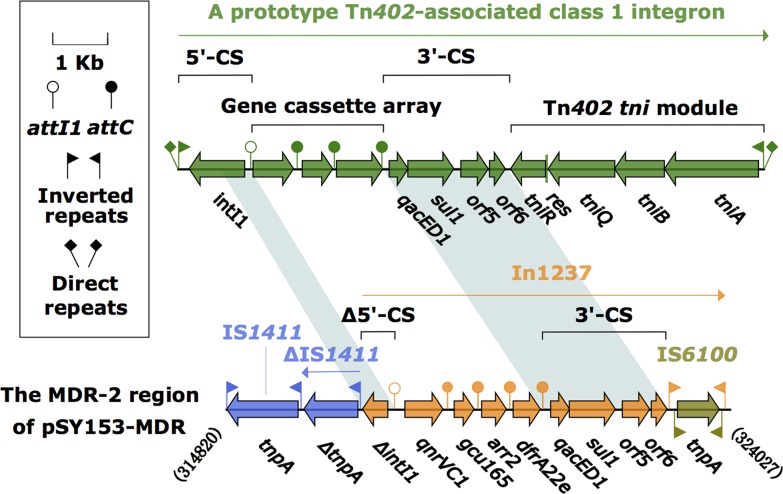
The MDR-2 region from pSY153-MDR, and comparison with related region Genes are denoted by arrows. Genes, mobile elements and other features are colored based on function classification. Shading denotes regions of homology (>95% nucleotide identity). Numbers in brackets indicate the nucleotide positions within pSY153-MDR.

The MDR-2 region was composed of a class 1 integron, In1237, and two copies of IS*1411*, one intact and the other truncated (Figure 2). In1237 carried a GCA consisting of *qnrVC1* (quinolones resistance)–*gcu165* (unknown function)–*arr2* (rifampicin resistance)–*dfrA22e* (dihydrofolate resistance). Notably, *dfrA22e* was a derivative of the reference *dfrA22* gene (accession number HM173356), containing variations Pro3Arg, Leu5Ser, Lys29Thr, Asn62Ser, Ser73Gly, Arg114His, Asp137Asn, and Glu138Ala. This *dfrA22e* cassette was also found in In1218 from *Aeromonas sobria* from ornamental fish (accession number KT315928). The expression of the In1237 cassette array was driven by a sole PcW promoter. Compared with the prototype class 1 integron, In1237 had undergone two major changes: i) loss of IRi and truncation of *intI1* through the connection of In1237 to ΔIS*1411*; and ii) replacement of *tni* by IS*6100*.

## MATERIALS AND METHODS

### Bacterial isolation and identification

The use of human specimens and all related experimental protocols was approved by the Committee on Human Research of the indicated institutions, and was carried out in accordance with the approved guidelines. Informed consent was obtained from the patient where indicated. Research involving biohazardous materials and all related procedures were approved by the Biosafety Committee of the National Institute for Communicable Disease Control and Prevention, Beijing, China. *P. putida* SY153 was isolated in 2012 from the urine specimen of a 68-year-old male patient with acute onset of cerebral infarction in a tertiary hospital in Sanya City, China. Bacterial species identification was performed by 16S rDNA gene amplification and sequencing [33]. The major plasmid-borne carbapenemase, extended-spectrum Δ-lactamase, and 16S rRNA methylase genes were screened by polymerase chain reaction (PCR) assays [34, 35]. All PCR amplicons were sequenced on an ABI 3730 Sequencer (Applied Biosciences, Foster City, CA, USA), using the same primers as for PCR, according to the manufacturer’s instructions.

### Plasmid conjugal transfer

Plasmid conjugal transfer experiments were carried out using sodium azide-resistant *Escherichia coli* J53 and colistin-resistant *P. aeruginosa* PAO1 (colistin MIC= 32 μg/mL) as the recipients, and SY153 as the donor. Aliquots (3 mL) of overnight culture of each donor and recipient strain were mixed, harvested, and resuspended in 80 μL of Brain Heart Infusion (BHI) broth (BD Biosciences). The suspensions were spotted on 1-cm^2^ hydrophilic nylon membrane filters with a 0.45-μm pore size (Millipore), which were then placed on BHI agar (BD Biosciences) plates and incubated at 25°C, 30°C, or 37°C for 12–18h. Bacteria were washed from the filter membranes and spotted onto Muller-Hinton (MH) agar plates containing 100 μg/mL sodium azide or 10 μg/mL colistin and 100 μg/mL ceftazidime for selection of *bla*_IMP-45_-positive *E. coli* or *P. aeruginosa* transconjugants.

### Detection of carbapenemase activity

Activity of class A/B/D carbapenemases in bacterial cell extracts was determined using a modified CarbaNP test [34]. Briefly, 2 mL of bacterial cultures with an optical density at 600 nm of 1.0–1.4 were harvested, washed, and resuspended in 500 μL of 20 mM Tris-HCl (pH 7.8), lysed by sonication, and pelleted by centrifugation. Aliquots (50 μL) of the supernatants were individually mixed with 50 μL of solutions I–V, followed by incubation at 37°C for a maximum of 2 h. The substrates in solutions I-V consisted of 0.054% phenol red, 0.1mM ZnSO_4_ (pH 7.8), with or without 0.6 mg/μL imipenem, 0.8 mg/μL tazobactam and/or 3 mM EDTA (pH 7.8).

### Antimicrobial susceptibility test

The antimicrobial susceptibility of the bacterial strains was determined by Etest (BioMérieux, Hazelwood, MO, USA), and interpreted as per Clinical and Laboratory Standards Institute guidelines [36].

### Sequencing and annotation

Genomic DNA was isolated from SY153 using a Wizard Genomic DNA Purification Kit (Promega, Madison, WI, USA). The genome was sequenced using a Single Molecule Real Time technique on a PacBio platform (Tianjin Biochip Corporation, Tianjin, China). A total of 87,287 polymerase reads, with a mean read length of 11,530 bp, were generated, resulting in a total of 1,006,456,227 bases with a 104-fold average coverage. The DNA contigs were assembled using HGAP 2.0 [37]. Open reading frames and pseudogenes were predicted using RAST 2.0 [38], combined with BLASTP/BLASTN [39] searches against the UniProtKB/Swiss-Prot [40] and RefSeq [41] databases. Annotation of resistance genes, mobile elements, and other features was carried out using CARD [42], ResFinder [43], ISfinder [44], INTEGRALL [45]. Multiple and pairwise sequence comparisons were performed using MUSCLE 3.8.31 [46] and BLASTN, respectively. Gene organization diagrams were drawn in Inkscape (https://inkscape.org).

### Nucleotide sequence accession number

The complete sequence of pSY153-MDR was submitted to GenBank under accession number KY883660.

## CONCLUSION

*bla*_IMP-45_ genes have previously been documented in both chromosomally- and plasmid-located In786 sequences from *P. aeruginosa* [29–31] and *S. maltophilia* isolates [29]. To date, only one *bla*_IMP-45_-carrying plasmid, namely pBM413, from *P. aeruginosa* has been fully sequenced. Data presented in the current study showed that *bla*_IMP-45_ has spread to *P. putida*, and this is the first report of determination of a fully-sequenced plasmid, carrying In786-borne *bla*_IMP-45_ gene, from *P. putida*.

*armA* genes have been reported in three *P. aeruginosa* isolates [31, 32], and in two of these isolates, *armA* is embedded in the Tn*1548*-associated regions and coexists with *bla*_IMP-45_ on a single plasmid [31]. The current study is the first report of *armA* in *P. putida*.

Coexistence of a large number of antibiotic resistance genes accounts for the extensive drug resistance of strain SY153, which is likely to be a reservoir of antimicrobial resistance genes. The presence of these determinants also aids in the survival of strain SY153 under different antimicrobial selection pressures. All of the resistance genes contained on plasmid pSY153-MDR are clustered in the two MDR regions, which have very complex mosaic structures. These regions can be dissected into various integrons, transposons, and transposon-like elements. Further study on the evolution and diversification of pSY153-MDR and related plasmids, including those without antibiotic resistance genes, is needed.

## SUPPLEMENTARY MATERIALS FIGURE AND TABLE



## Abbreviations

MDR: multidrug-resistant
MBLs: Metallo-β-lactamases
ORFs: open reading frames
DRs: direct repeats
IRL: inverted repeat left
IRR: inverted repeat right
IRi: inverted repeat at the integrase end
CS: conserved segment
GCA: gene cassette array
IRt: inverted repeat at the *tni* end
IS: insertion sequence
MIC: minimum inhibitory concentration
BHI: Brain Heart Infusion
