# Supplementary material for: pSY153-MDR, a p12969-DIM-related mega plasmid carrying blaIMP-45 and armA, from clinical Pseudomonas putida
Source: Oncotarget. 2017 Jul 22;8(40):68439–47. doi: 10.18632/oncotarget.19496 (PMC5620268; doi:10.18632/oncotarget.19496)
Supplement: Supplementary file 1 [file oncotarget-08-68439-s001.pdf]

pSY153-MDR, a p12969-DIM-related mega plasmid carrying *bla*$_{IMP-45}$ and *armA*, from clinical *Pseudomonas putida*

SUPPLEMENTARY MATERIALS

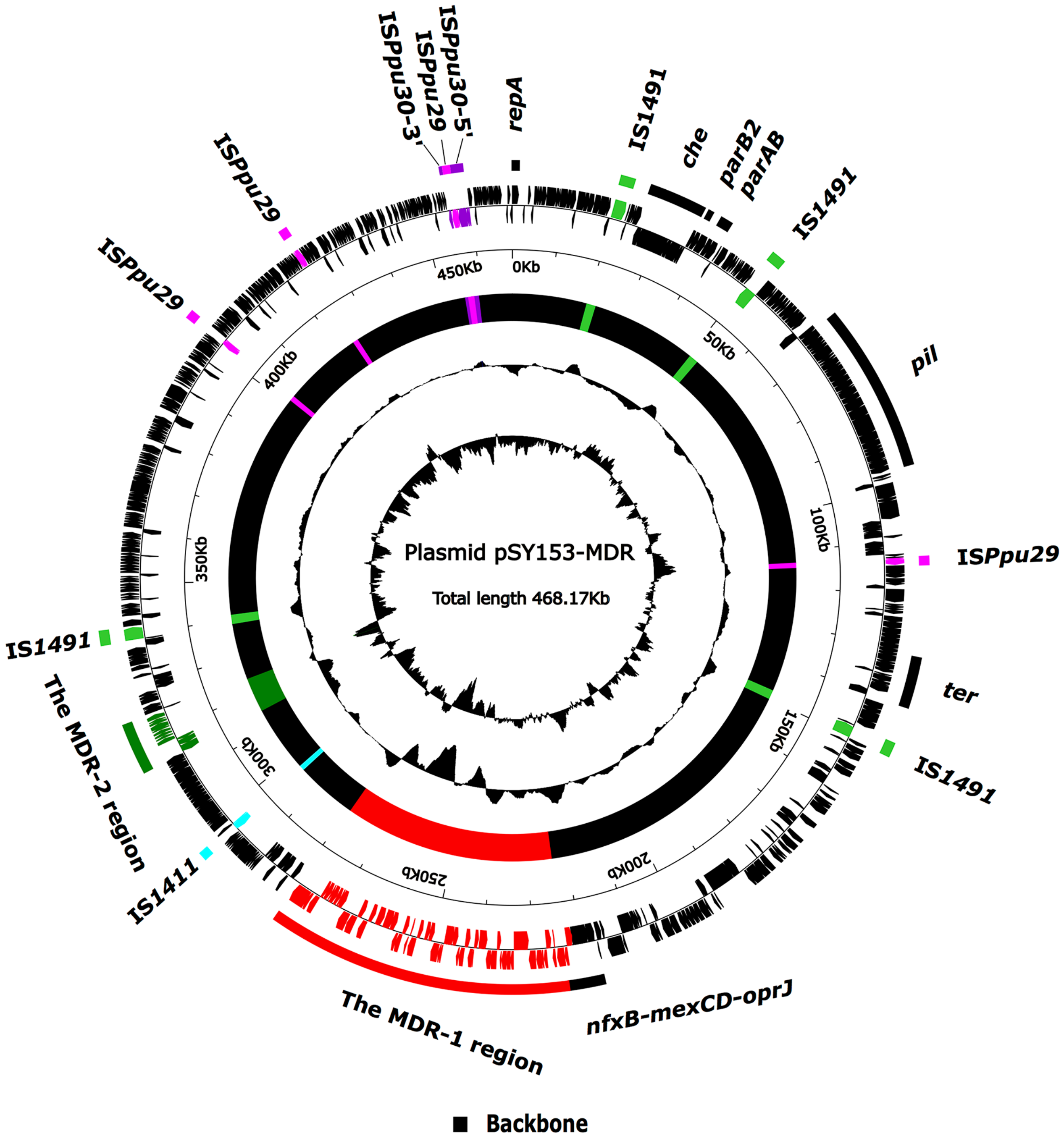

Supplementary Figure 1: Schematic map of pSY153-MDR. Genes are denoted by arrows, and the backbone and accessory module regions are highlighted in black and grey, respectively. The innermost circle presents GC-skew [(G-C)/(G+C)], with a window size of 500 bp and a step size of 20 bp. The next-to-innermost circle presents GC content.

Supplementary Table 1: Antimicrobial drug susceptibility profiles for *P. putida* SY153

Category	Antibiotics	MIC (μg/mL)/antimicrobial susceptibility
Penicillins	Piperacillin	≥256R
	Piperacillin/tazobactam[a]	≥256R
Cephalosporins	Ceftazidime	≥256R
	Cefepime	≥256R
	Cefotaxime	≥256R
	Ceftriaxone	≥256R
Carbapenems	Imipenem	≥32R
	Meropenem	≥32R
Monobactam	Aztreonam	16 I
Aminoglycosides	Gentamicin	≥256R
	Amikacin	≥256R
Tetracycline	Tetracycline	≥256R
Fluoroquinolones	Ciprofloxacin	≥32R
	Levofloxacin	≥32R
Folate pathway Inhibitors	Trimethoprim/sulfamethoxazole[b]	≥32R
Phenicol	Chloramphenicol	≥32R
Macrolides	Erythromycin[c]	≥256
	Azithromycin[c]	≥256
Rifampin	Rifampin[c]	≥32
Nitrofuran	Nitrofurantoin[c]	≥512

S=sensitive; R=resistant; I= intermediate.

[a] Piperacillin/tazobactam: tazobactam at a fixed concentration of 4 μg/mL.

[b]Trimethoprim/sulfamethoxazole: a 1:19 combination.

[c]"R" and "S" cannot be judged because interpretive criteria for corresponding antibiotics are currently lacking for *P. putida*.
